# Tritium-Labeled Compounds VI. Alditols-*1-t* and Alditols-*2-t*^1^

**DOI:** 10.6028/jres.064A.044

**Published:** 1960-10-01

**Authors:** Harriet L. Frush, Horace S. Isbell, Alexander J. Fatiadi

## Abstract

By reduction of aldoses and aldonic lactones with lithium borohydride-*t*, the following 1-tritium-labeled alditols were prepared: d-arabinitol-*1-t*, d-lyxitol-*1-t*, d-ribitol-*1-t*, d-xylitol-*1-t*, d-galaetitol-*1-t*, d-mannitol-*1* (*6*)-*t*, d-glucitol-*1-t*, d-talitol-*1-t*, l-gulitol-*1-t* (d-glucitol *6-t*), l-rhamnitol-*1-t*, d-*glycero*-d-*gulo*-heptitol-*1-t*, and 4-*O-β*-d-galactopyranosyl-d-glucitol-*1-t*. By reduction of ketoses with lithium borohydride-*t*, the following epimeric pairs of 2-labeled alditols were prepared and subsequently separated: d-mannitol-*2*(*5*)-*t* and d-glucitol-*2-t*; l-gulitol-*2-t* (d-glucitol-5-*t*) and l-iditol-*2-t*; d-galactitol-2-*t* and d-talitol-*2-t*; d-*glycero*-d-*gulo*-heptitol-*2-t* and d-*glycero*-d-*ido*-heptitol-*2-t*; and d-*glycero-B-galacto*-heptitol-*2-t* and d-*glycero*-d-*talo*-heptitol-*2-t*.

The yields of the epimeric alditols formed from ketoses were determined by an isotopedilution technique. Stereomeric relationships are discussed for the labeled alditols and for the ketoses derivable from them by oxidation with *Acetobacter suboxydans*.

## 1. Introduction and Discussion

Tritium-labeled alditols are useful intermediates for synthesizing tritium-labeled ketoses and for studying a wide variety of chemical and biological reactions. As part of a program on the development of methods for synthesizing tritium-labeled carbohydrates [1, 2, 3, 4, 5, 6],^2^ procedures have now been developed for preparing alditols position-labeled with tritium.

Nonradioactive sodium borohydride has been used for reducing aldoses [7] and lactones [8,9] to alditols. However, for the preparation of tritium-labeled materials, it was considered advantageous to use lithium borohydride-*t* (instead of the sodium analog), because it may be more easily prepared.^3^ Hence, processes were developed for the use of lithium borohydride-*t* in preparing tritium-labeled alditols.

The experimental conditions under which lithium borohydride is used are critical, insofar as the extent and efficiency of the reduction are concerned. In the previous preparation of aldoses-*1-t* by the reduction of aldonic lactones [4], lithium borohydride-*t*, dissolved in anhydrous pyridine, was added to a solution of the lactone in water. The use of pyridine as a solvent avoids decomposition of the hydride and appears to suppress the further reduction of the aldose to the alditol. However, in the preparation of labeled alditols, better yields were obtained when tetrahydrofuran, instead pyridine, was used as the solvent.

Alditols may be prepared by the reduction of aldoses, aldonic lactones, or ketoses. Aldoses and lactones, on reduction, form alditols having, respectively, one and two atoms of hydrogen-*t* at C1. Thus, the product derived from the lactone has twice the specific activity of that derived from the aldose. Ketoses, on reduction, form epimeric pairs of alditols having one atom of hydrogen-*t* at C2; subsequent separation of the alditols is necessary.

Tables 1, 2, and 3 summarize the results obtained by the reduction of aldoses, aldonic lactones, and ketoses, respectively, to alditols. Yields of the alditols were determined by (a) radioactivity assay of the purified solutions and (b) isotopedilution techniques. Table 3 gives the yields of the epimeric alditols formed from several ketoses, a subject of considerable theoretical interest.

Tritium-labeled products having high activities are subject to decomposition from self-radiation, and hence should not be held long in storage. The activities of the products listed in tables 1, 2, and 3 are adequate for most purposes, and decomposition over the course of several months has been slight. Position-labeled products of higher activity can be made, but these must be used within a relatively short time.

## 2. Nomenclature of Position-Labeled Alditols and Related Ketoses

The presence of an isotopic atom at a definite position in the molecule of an alditol gives rise to certain problems of nomenclature. An alditol that has *no axis or plane of symmetry* is related to two aldoses. If this alditol is position-labeled, the position of the label is designated differently in the two names. For example, the alditol obtained by reducing d-glucose-*1-C*^14^ may be named either d-glucitol-*1-C*^14^ or l-gulitol-*6-C*^14^.

An unlabeled alditol that has a *plane of symmetry* is a *meso* compound derivable from either the d or the l form of an unlabeled aldose. However, this alditol is truly asymmetric if position-labeled, and is classified as d or l according to the configuration and the position of the label. Thus, d-galactitol-*1-t* is enantiomorphic with l-galactitol-*1-t*, but is identical with l-galactitol-*6-t*; similarly, d-xylitol-*1-C*^14^ may also be named l-xylitol-5-*C*^14^.

An alditol that has an *axis of symmetry* is related to only one aldose. Because the two parts of the molecule are identical, the corresponding atoms or groups are indistinguishable. For instance, in d-mannitol, the structure and configuration are the same at Cl and C6 (as well as at C2 and C5, and at C3 and C4). Hence, if the alditol is labeled in one position of the molecule, it is labeled also in the corresponding position. Thus, for example, the alditol obtained by reducing d-mannose-*1-C*^14^ is d-mannitol-*1*(*6*)-*C*^14^.

Certain of the alditols are oxidized to ketoses by *Acetobacter suboxydans*. This organism oxidizes a compound containing the structure 
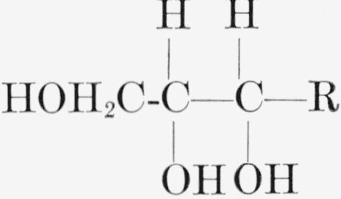
 by converting the group at the penultimate carbon atom to 
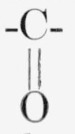
 [11]. Table 4 lists the labeled ketoses that can be produced by *A. suboxydans* from 1-tritium-labeled alditols having six or fewer carbon atoms. d-Fructose-*1* (*6*)-*t* and l-sorbose-*6-t* have already been prepared. The latter sugar is an intermediate for the preparation of l-ascorbic-*6-t* acid.

## 3. Apparatus and Materials

The reductions were conducted in a closed system, in 50-ml flasks each having a rubber-capped side-arm for the injection of liquids. The flasks were attached to a vacuum manifold, which was part of the equipment previously described for collecting and handling tritium gas [3].

Lithium borohydride-*t* was prepared from non-radioactive lithium borohydride by hydrogen-tritium exchange. Solutions of lithium borohydride-*t* in dry tetrahydrofuran were then prepared by the procedure given in ref [3].

Radioactivity assays were made with a 2π, window-less, gas-flow, proportional counter. Materials with low activity were counted in films of sodium *O*-(carboxymethyl) cellulose on 2-in. planchets [2]. Those with high activity were assayed as solutions in formamide. The procedure was essentially the same as that developed for the assay of carbon-14 [12], but was standardized with a sample having known tritium content. The counting efficiency is extremely low, but the precision of the method is excellent. Under the conditions used, one count per second is equivalent to 0.128 *μ*c of tritium.

## 4. Procedures

### 4.1. Preparation of Alditols-*1-t* From Aldoses

A magnetic stirring bar and 4 millimoles of the aldose to be reduced were placed in a 50-ml reaction flask equipped with a rubber-capped side-arm. The flask was attached to the vacuum manifold and evacuated. The connection of the reaction flask to the manifold was closed, and the flask was cooled in a shallow ice-bath resting on a magnetic stirrer.

Five milliliters of ice-water containing 1 millimole (106 mg) of sodium carbonate was injected through the rubber cap by means of a hypodermic needle and syringe. The stirrer was started, and 2 ml of a solution containing 1.0 millimole (22 mg) of lithium borohydride-*t* in dry tetrahydrofuran was added by needle through the rubber cap; stirring was continued for 15 minutes. The solution was allowed to stand at room temperature for several hours (or overnight) and was then frozen in liquid nitrogen. The by-product hydrogen-*t*, formed by reaction of the lithium borohydride-*t* with water, was transferred to the manifold and either stored in a flask or converted to water-*t*. Finally, the connection to the manifold was closed and the flask was removed. The solvent (water and tetrahydrofuran) was evaporated in a rotary vacuum still equipped with a trap immersed in a dry-ice freezing bath. Water was added and the solution was again concentrated in the still; addition of water and concentration were repeated several times. Ultimately, the distillate in the trap was discarded as radioactive waste. The residue in the flask was dissolved in water, and the solution was passed through a column containing 10 ml of a cation-exchange resin. The effluent was evaporated to about 1 ml in the vacuum still; then, about 15 ml of methanol was added, and the solvent was again evaporated. Addition of methanol and evaporation were repeated several times in order to remove all boric acid as methyl borate. An aqueous solution of the residue was passed through a small column of mixed cation- and anion-exchange resins, and the effluent^4^ was concentrated under reduced pressure. The residue was crystallized from hot ethanol or other suitable solvent, and the specific activity of the product was determined as described in section 3 and ref [2]. The alditol-*1-t* was recrystallized until the specific activity became constant.

### 4.2. Preparation of Alditols-*1-t* From Aldonic Lactones

The procedure for preparing 1-tritium-labeled alditols from aldonic lactones was the same as that described in section 4.1, except for the following changes: (a) 2 millimoles of the aldonic lactone were reduced in place of 4 millimoles of the aldose; (b) sodium carbonate was omitted in the reduction step;^5^ and (c) 1.25 millimoles of lithium borohydride-*t* were used in place of 1 millimole.

### 4.3. Preparation of Alditols-*2-t* From Ketoses

The method for reducing ketoses with lithium borohydride-*t* was essentially the same as that given in section 4.1 for reducing aldoses. Two millimoles of the ketose were reduced with 0.5 millimole of lithium borohydride-*t* having an activity of approximately 9 mc per milliatom of hydrogen. The product was then treated as follows:

- The yields of the separate epimeric alditols-*2-t* were determined by an isotope-dilution technique. Aliquots of the solution containing approximately 5 *μ*c of tritium were diluted with 100 mg of the nonradioactive alditol under investigation. The alditol carrier was then recrystallized repeatedly from a suitable solvent, ordinarily ethanol, until a product of constant activity was obtained. From the relative size of the aliquot used, the weight of the carrier taken, and the specific activity of the carrier after recrystallization, the total activity of the alditol in the parent solution was readily calculated.
- After removal of aliquots for analysis, the solution was concentrated and the epimeric alditols were separated by fractional crystallization, usually from ethanol. In most cases, one of the alditols crystallized more readily than the other; satisfactory separations were obtained by seeding the sirup with one epimer and removing the crystals of the substance before crystals of the other appeared. In some cases, the products were separated by addition of the nonradioactive alditol as carrier. The identity and purity of the alditol-*2-t* were confirmed by the following isotope-dilution technique:

A 1-mg sample of the purified alditol-*2-t* of known radioactivity was diluted with 100 mg of the pure, nonradioactive alditol. The carrier mixture was recrystallized three times, and the product was assayed for radioactivity. If A and B were, respectively, the specific activities of the alditol-*2-t* and the carrier mixture, then the purity of the alditol-*2-t* (in percent) was (101B/A)×100. All of the alditols reported in table 3 gave results within 4 percent of the expected value.

## Figures and Tables

**Table 1:** Reduction of aldoses with lithium borohydride-*t*^a^

Aldose	Alditol-*1-t*	Specific activity	Radiochemical yield
			
		*μc/mg*	%
d-Arabinose	d-Arabinitol-1-*t*	59.8	94
d-Lyxose	d-Lyxitol-*1-t* (d-Arabinitol-*5-t*)	59.8	71
d-Ribose	d-Ribitol-*1-t*	59.8	80
d-Xylose	d-Xylitol-*1-t*	60.0	86
d-Galactose^b^	d-Galactitol-*1-t*	44	90
d-Glucose	d-Glucitol-*1-t*	50.0	81
d-Talose	d-Talitol-*1-t*	50.5	67

a Experimental details are given in section 4.1.

b Preparation reported earlier in ref [3]. The lithium borohydride had a specific activity different from that of the reductant used in other preparations.

**Table 2:** Reduction of aldonic lactones with lithium borohydride-*t*^a^

Lactone	Alditol-*1-t*^b^	Radiochemical yield
		
		%
d-Arabono-*γ*-	d-Arabinitol-*1-t*	79.2
d-Xylono-*γ*-	d-Xylitol-*1-t*	80.0
d-Galactono-*γ*-	d-Galactitol-*1-t*	71.5
d-Glucono-*δ*-	d-Glucitol-*1-t*	70.8
d-Glucono-*γ*-	d-Glucitol-*1-t*	70.3
l-Gulono-*γ*-	l-Gulitol-*1-t* (d-glucitol-*6-t*)	66.6
d-Mannono-*γ*-	d-Mannitol-*1* (6)-*t*^c^	76.6
l-Rhamnono-*γ*-	l-Rhamnitol-*1-t*	75.5
d-*glycero*-d-*gulo*-Heptono-*γ*-	d-*glycero*-d-*gulo*-Heptitol-*1-t*	67.4
Lactobiono-*γ*-	4-*O-β*-d-Galactopyranosyl-d-glucitol-*1-t*	61.3

a Experimental details are given in section 4.2.

b The products had an activity of approximately 19 millicuries per millimole.

c The preparation of d-mannitol-*1* (*6*)-*t* from 2,3:5,6-di-*O*-isopropylidene-d-mannofuranose was described in an earlier publication [3].

**Table 3:** Reduction of ketoses with lithium borohydride-*t*^a^

Ketose	Alditol-*2-t*	Radiochemical yield^b^
		
		%
d-Fructose	d-Mannitol-2(*5*)-*t*	43.2
	d-Glucitol-*2-t*	42.1
l-Sorbose	l-Gulitol-*2-t*	27.9
	l-Iditol-2(*5*)-*t*	
d-Tagatose	d-Galactitol-*2-t*	22.9
	d-Talitol-*2-t*	59.5
d-*gluco*-Heptulose	d-*glycero*-d-*gluco*-Heptitol-*2-t*	22.6
	d-*glycero*-d-*ido*-Heptitol-*2-t*	32.1
d-*manno*-Heptulose	d-*glycero*-d-*galacto*-Heptitol-*2-t*	33.2
	d-*glycero*-d-*talo*-Heptitol-*2-t*	60.0

a Experimental details are given in section 4.3.

b Yields were determined by isotope dilution.

**Table 4:** Tritium-labeled alditols and ketoses derivable from aldoses^a^

Aldoses	Alditols-*t*	Ketoses-*t*^b^,^c^
		
d-Glycerose	d-Glyceritol-*1-t* (l-glyceritol-*3-t*)	Dihydroxyacetone-*1* (*3*)-*t*.
l-Glycerose	l-Glyceritol-*1-t* (d-glyceritol-*3-t*)	Dihydroxyacetone-*1* (*3)-t*
d-Erythrose	d-Erythritol-*1-t* (l-erythritol-*4-t*	l-*glycero*-Tetrulose-*4-t*.
l-Erythrose	l-Erythritol-1-*t* (d-erythritol-*4-t*)	l-*glycero*-Tetruiose-*1-t*.
d-Threose	d-Threitol-*1*(*4*)-*t*	
l-Threose	l-Threitol-*1* (*4*)-*t*	
d-Arabinose	d-Arabinitol-*1-t* (d-lyxitol-*5-t*)	d-*threo*-Pentulose-*5-t*.
l-Arabinose	l-Arabinitol-*1-t* (l-lyxitol-*5-t*)	
d-Lyxose	d-Lyxitol-*1-t* (d-arabinitol-*5-t*)	d-*thero*-Pentulose-*1-t*.
l-Lyxose	l-Lyxitol-*1-t* (l-arabinitol-*5-t*)	
d-Ribose	d-Ribitol-*1-t* (l-ribitol-*5-t*)	l-*erythro*-Pentulose-*5-t*.
l-Ribose	l-Ribitol-*1-t* (d-ribitol-*5-t*)	l-*erythro*-Pentulose-1-*t*.
d-Xylose	d-Xylitol-*1-t* (d-ribitol-*5-t*)	
d-Xylose	l-Xylithol-*1-t* (d-ribitol-*5-t*)	
d-Allose	d-Allitol-*1-t* (l-allitol-*6-t*)	l-Psicose-*6-t*.
l-Allose	l-Allitol-*1-t* (d-allitol-*6-t*)	l-Psicose-*1-t*.
d-Altrose	d-Altritol-*1-t* (d-talitol-*6-t*)	d-Tagatose-*6-t*.
l-Altrose	l-Altritol-*1-t* (l-talitol-*6-t*)	
d-Galactose	d-Galactitol-*1-t* (l-galactitol-*6-t*)	
l-Galactose	l-Galactitol-*1-t* (d-galactitol-*6-t*)	
d-Glucose	d-Glucitol-*1-t* (l-gulitol-*6-t*)	l-Sorbose-*6-t*.
l-Glucose	l-Glucitol-*1-t* (d-gulitol-*6-t*)	
d-Gulose	d-Gulitol-*1-t* (l-glucitol-*6-t*)	
l-Gulose	l-Gulitol-*1-t* (d-glucitol-*6-t*)	l-Sorbose-*1-t*.
d-Idose	d-Iditol-*1*(*6*)-*t*	
l-Idose	l-Iditol-*1*(*6*)-*t*	
d-Mannose	d-Mannitol-*1*(*6*)-*t*	d-Fructos-1(*6*)-*t*.
l-Mannose	l-Mannitol-*1*(*6*)-*t*	
d-Talose	d-Talitol-*1-t* (d-altritol-*6-t*)	d-Tagatose-*1-t*.
l-Talose	l-Talitol-*1-t* (l-altritol-*6-t*)	

a Relationships are illustrated for compounds having six or fewer carbon atoms.

b Ketoses derivable from sterically suitable alditols, through oxidation by *Acetobacter suboxydans*. The other alditols listed do not have the requisite configuration for attack by *A. suboxydans*.

c Systematic names are as follows: l-*ribo*-hexulose(l-psicose); d-*lyxo*-hexulose (d-tagatose) ; l-*xylo*-hexulose(l-sorbose); and d-*arabino*-hexulose (d-fructose).

## Footnotes

2 Figures in brackets indicate the literature references at the end of this paper.

3 These materials can be prepared by tritium-hydrogen exchange, which occurs at about 200° C for lithium borohydride and at about 350° C for sodium borohydride [10]. The preparation of lithium borohydride-*t* is described in ref [3].

4 The effluent, when tested with a conductivity meter, showed the absence of ionic impurities.

5 Reduction of aldonic lactones to alditols is a two-step process, in the first of which the aldose is formed. Although an alkaline medium facilitates reduction of the aldose to the alditol, alkali tends to convert the lactone to the nonreducible salt. Hence, it was omitted in this reduction.
